# Supplementary material for: The Systems Biology Research Tool: evolvable open-source software
Source: BMC Syst Biol. 2008 Jun 29;2:55. doi: 10.1186/1752-0509-2-55 (PMC2446383; doi:10.1186/1752-0509-2-55)
Supplement: Additional file 1 — SBRT Archive. An archive of the current version of the Systems Biology Research Tool. [file 1752-0509-2-55-S1.zip › sbrt-1.4.0/doc/developers_guide/api/sbrt/kernel/math/stat/comp/class-use/TauResult.html]

Uses of Class sbrt.kernel.math.stat.comp.TauResult


|  |  |  |
| --- | --- | --- |
| **Overview** \| **Package** \| **Class** \| **Use** \| **Tree** \| **Deprecated** \| **Index** \| **Help** | |  |
| PREV   NEXT | **FRAMES**    **NO FRAMES**     **All Classes** |  |


---


## **Uses of Class sbrt.kernel.math.stat.comp.TauResult**

| Packages that use TauResult | |
| --- | --- |
| **sbrt.kernel.math.stat.comp** | Provides classes and interfaces for performing statistical computations. |
| **sbrt.shell.mng.stat** | Provides classes and interfaces for creating statistical process managers. |
| **sbrt.shell.text.math** | Provides classes and interfaces for defining formats for mathematical objects. |

Uses of TauResult in sbrt.kernel.math.stat.comp

| Methods in sbrt.kernel.math.stat.comp that return TauResult | |
| --- | --- |
| `TauResult` | `KendallTau.corr(java.util.Collection<java.lang.Double> xValues, java.util.Collection<java.lang.Double> yValues)`             Computes and returns the correlation results for the provided values. |
| `TauResult` | `KendallTau.corr(double[] xValues, double[] yValues)`             Computes and returns the correlation results for the provided values. |
| `TauResult` | `KendallTau.corr(java.lang.Double[] xValues, java.lang.Double[] yValues)`             Computes and returns the correlation results for the provided values. |
| `TauResult` | `KendallTau.corr(java.util.Map<? extends V,java.lang.Double> xValues, java.util.Map<? extends V,java.lang.Double> yValues)`             Computes and returns the correlation results for the provided values. |

Uses of TauResult in sbrt.shell.mng.stat

| Methods in sbrt.shell.mng.stat that return types with arguments of type TauResult | |
| --- | --- |
| `CorrelationAnalysis<java.lang.String,TauResult>` | `KendallTauManager.getProcess()`             Returns the process controlled by this process manager. |

Uses of TauResult in sbrt.shell.text.math

| Methods in sbrt.shell.text.math with parameters of type TauResult | |
| --- | --- |
| `java.lang.String` | `CorrResFormatterV1.format(TauResult results)`             Returns a formatted string representation of the provided correlation results. |

---


|  |  |  |
| --- | --- | --- |
| **Overview** \| **Package** \| **Class** \| **Use** \| **Tree** \| **Deprecated** \| **Index** \| **Help** | |  |
| PREV   NEXT | **FRAMES**    **NO FRAMES**     **All Classes** |  |


---
